# Comprehensive Study of Intermediate and Critical Quality Attributes for Process Control of High-Shear Wet Granulation Using Multivariate Analysis and the Quality by Design Approach

**DOI:** 10.3390/pharmaceutics11060252

**Published:** 2019-06-01

**Authors:** Jong Kwon Han, Beom Soo Shin, Du Hyung Choi

**Affiliations:** 1 School of Pharmacy, Sungkyunkwan University, Suwon 440-746, Korea; jongkwon@skku.edu; 2 Department of Pharmaceutical Engineering, Inje University, Gyeongnam 621-749, Korea

**Keywords:** quality by design, scale-up strategy, critical process parameters, intermediate quality attributes, high-shear wet granulation, multivariate analysis

## Abstract

A robust manufacturing process and the relationship between intermediate quality attributes (IQAs), critical quality attributes (CQAs), and critical process parameters (CPPs) for high-shear wet granulation was determined in this study. Based on quality by the design (QbD) approach, IQAs, CQAs, and CPPs of a telmisartan tablet prepared by high-shear wet granulation were determined and then analyzed with multivariate analysis (MVA) to evaluate mutual interactions between IQAs, CQAs, and CPPs. The effects of the CPPs on the IQAs and CQAs were quantitatively predicted with empirical models of best fit. The models were used to define operating space, and an evaluation of the risk of uncertainty in model prediction was performed using Monte Carlo simulation. MVA showed that granule size and granule hardness were significantly related to % dissolution. In addition, granule FE (Flow Energy) and Carr’s index had effects on tablet tensile strength. Using the manufacture of a clinical batch and robustness testing, a scale-up from lab to pilot scale was performed using geometric similarity, agitator torque profile, and agitator tip speed. The absolute biases and relative bias percentages of the IQAs and CQAs generated by the lab and pilot scale process exhibited small differences. Therefore, the results suggest that a risk reduction in the manufacturing process can be obtained with integrated process parameters as a result of the QbD approach, and the relationship between IQAs, CQAs, and CPPs can be used to predict CQAs for a control strategy and SUPAC (Scale-Up and Post-Approval Guidance).

## 1. Introduction

Pharmaceutical manufacturing processes are complex and controlled by multi-factorial relationships between materials and process parameters. Therefore, it is difficult to investigate the pharmaceutical manufacturing process with the OFAT (one-factor-at-a-time) method. Combination approaches, such as experimental design and MVA, could present a comprehensive and intensive understanding of the relevant multi-factorial relationships between process parameters and product quality attributes [1,2]. MVA is the statistical method that can reduce or classify variables using correlation between variables. It also provides visual effects for multivariate data to enable an understanding of the relationship between distribution features, patterns, natural groupings, and relationships variables [1,3,4,5]. In pharmaceutical experimental design, there are numerous variables, such as material properties, manufacturing processes, quality attributes, and intermediate quality attributes, which occur during the manufacturing process. It is difficult to study the relationships among such a large number of variables because only a limited number of variables can be processed through experimental design analysis [1]. Generally, the number of control factor is less than 3–4, and the level of control factors is more than 3 levels in the optimization stage. If the number of control factors increases or the number of level increases, the number of experiment increases exponentially [6]. In experimental design, the relationship between control factors and response factors is statistically analyzed with analysis of variance. However, it is difficult to define the relationship between response factors using experimental design. MVA can be regarded as a complementary method to experimental design, which presents additional information about the product and processes, such as the effect of intermediate quality attributes on product quality attributes. Therefore, when MVA and experimental design are combined, the integrated MVA is a more powerful tool to elucidate the complex relationships in the pharmaceutical manufacturing process.

High-shear wet granulation is the most commonly used technique in the pharmaceutical industry because of various advantages, such as lower use of binding agents, shortened processing times, the formation of reproducible granules with a uniform size distribution, and formation of dense granules suitable for tableting [7]. Agitator speed, chopper speed, agitator shape, chopper shape, liquid to solid ratio, liquid addition rate, massing time, drying temperature, and drying time could be considered process parameters of high-shear wet granulation. The process parameters have an effect on product quality attributes (e.g., dissolution profile, content uniformity, assays, and tablet hardness, among others), as well as intermediate quality attributes (e.g., granule size, granule hardness, granule flow energy, and granule density, among others) [8,9,10,11,12,13]. Improper agitator and chopper speeds can result in uncontrolled granule growth [8,13,14,15,16]. This results in undesired granule porosity and granule strength. In addition, a high agitator speed presents a high shearing force to powder bed, which might produce smaller granules with narrow size distribution [17,18]. Wet massing time might be one of the most important parameters in the granulation process because the parameter allows the coalescence and consolidation of granules by kneading the wet mass [8,19,20]. The wet massing time should be optimized to obtain desired granule properties. The failure of massing time control can produce weak granules or undesired density granules, which makes them difficult to handle for next unit operation. Moreover, the wet massing time may have an effect on granule size and size distribution. Improper wet massing time may produce a bimodal granule size distribution or monomodal granule size distribution [12,21,22]. Additionally, the liquid addition rate is related to the onset of the nucleation stage and operation time [9]. The liquid is uniformly distributed on a powder bed at a slow addition rate. This might decrease the powder agglomeration time. On the contrary to this, a fast addition rate may not present uniform distribution of the liquid on a powder bed, which leads to lump formation with a wide granule size distribution [23,24,25,26]. The improper process can result in undesirable intermediate quality attributes, which could be significantly related to poor product quality attributes. 

Based on quality by the design (QbD) implementation and various studies of the QbD approach, in the early stage of development, the formulation and the processes are, in general, optimized using lab-scale equipment. Subsequently, the formulation is fixed during the scale-up [27,28,29,30]. Thus, the formulation should be robust and has to lead to the same quality of product using the lab-scale and pilot or commercial-scale equipment. Therefore, the scale-up process should be significantly controlled to present a target quality for the product. Although there are various scale-up strategies, general considerations in the scale-up process are dimensional analysis and the principle of similarity [28,31,32]. Dimensional analysis is based on the assumption that a mathematical formulation of a physical process should be valid in any system of dimensions. It is a tool to produce dimensionless numbers that completely describe the process. The dimensionless numbers most commonly used to describe the high-shear wet granulation are Newton, Froude, and Reynolds. The Newton number is related to the drag force acting on the agitator and quantified power required to overcome friction in fluid flow in a stirred vessel. The Froude number is the interplay of the centrifugal force and the centripetal force produced by the wall. The Reynolds number is related to the inertial force and the viscous force [28,31,32]. The principle of similarity can be explained with geometrical similarity, kinematic similarity, and dynamic similarity. The dimensionless numbers might have the same numerical value under the condition of similarity. However, the scale-up strategy of high-shear wet granulation is particularly difficult and often problematic because of the inherent heterogeneous nature of the materials used and the various process parameters are complexly interrelated. When a scale-up process of high-shear wet granulation is not well performed, the IQAs might change. A change in the granule size, granule size distribution, granule density, compressibility, or granule flow energy might significantly affect product quality, such as tablet hardness, dissolution, content uniformity, disintegration time, tablet friability, and assays [33]. If the improper scale-up of high-shear wet granulation is not detected at the end product test stage, it could result in huge economic losses and wasted time. 

Although the QbD approach is essentially applied for the development of pharmaceutical products, most studies of QbD approach for the development of pharmaceutical products were focused on the relationships between control factors (critical material attributes or CPPs) and response factors (drug product CQAs). In addition, IQAs after unit operation might not be significantly investigated to define the relationship with drug product CQAs in the QbD approach [33,34,35,36,37,38,39]. This study was conducted to investigate the robust manufacturing process with respect to the relationships among IQAs, critical quality attributes (CQAs), and critical process parameters (CPPs) for high-shear wet granulation. Based on the QbD approach, the control and response factors were determined by risk assessment, which is based on our knowledge and experience with related processes and the target profiles of the control tablet. The model drug was telmisartan, and a response surface design was used to obtain the optimal process parameters of high-shear wet granulation to prepare telmisartan granules using three control factors: agitator speed, massing time, and binding suspension addition rate. The quantitative effects of the different control factors on the response factors were accurately predicted using empirical models of best fit. The models and Monte Carlo simulation were used to identify the operating space. The relationships between IQAs and CQAs were analyzed with principal component analysis (PCA), where partial least squares (PLS) was used to evaluate the relationships among IQAs, CQAs, and CPPs. To manufacture a clinical batch and conduct robustness testing, a scale-up from lab to pilot scale was performed using geometric similarity, agitator torque profile, and agitator tip speed. Moreover, a pharmacokinetics study was performed with the clinical batch. 

## 2. Materials and Methods

### 2.1. Materials

Telmisartan, D-mannitol, meglumine, and magnesium stearate were received from Jeil Pharmaceutical Co., Ltd. (Seoul, Korea). Polyvinylpyrrolidone (PVP K25) and crospovidone (Kollidon^®®^ CL-F) were obtained from BASF AG (Ludwigshafen, Germany). Dicalcium phosphate and sodium hydroxide were obtained from Sigma-Aldrich Co. (St. Louis, MO, USA). Microcrystalline cellulose (MCC 101) was obtained from DFE Pharma (Dusseldorf, Germany). F-MELT^®®^ (Type C) was obtained from Fuji Chemical Industries Co., Ltd. (Toyama, Japan). All other reagents were of analytical or HPLC grade and were used as received.

### 2.2. Risk Assessment

IQAs, CQAs, and CPPs were established by risk assessment, which was based on our knowledge and experience with the related process and the target profiles of the control tablet. Failure mode and effects analysis (FMEA) was applied to quantify the degree of risk connected with these process parameters [34,36]. Based on the total risk priority number (RPN), a score matrix presented the priority and/or level of attention required to be applied to each step. Risk quantification was performed by ranking severity (S), probability (P), and detectability (D), which were rated individually from 1 to 5. Severity was related to the results of the failure mode and considered the worst result of the failure, determined by the degree of effect to repair the failure. The likelihood of failure was represented as the probability and detectability, which indicated the ability to determine and detect the existence of a failure mode, respectively. The RPN was calculated by multiplying the three scoring columns [34]. RPN was classified as low risk (1–45), medium risk (46–90), and high risk (91–125). Moreover, a pareto chart was combined with the RPN to identify the control factors, which were evaluated using the experimental design. 

### 2.3. Experimental Design to Investigate High-Shear Wet Granulation in Lab Scale

To generate the experimental design, Design-Expert^®^ software (version 10; Stat-Ease Inc., Minneapolis, MN, USA) was used. Response surface design was used to identify the optimal process parameters of high-shear wet granulation to prepare telmisartan granules. The three control factors were agitator speed (*p*_1_), massing time (*p*_2_), and binding suspension addition rate (*p*_3_). IQAs, such as granule hardness (*q*_1_), Carr’s index of granules (*q*_2_), granule size (*q*_3_), granule true density (*q*_4_), granule bulk density (*q*_5_), and granule FE (*q*_6_), and CQAs, such as tablet tensile strength (*q*_7_) and % dissolution (*q*_8_–*q*_11_) were evaluated as responses of experimental design, which were determined during the risk assessment. The best-fit empirical model was used to predict the relationship between control factors and response factors by comparing statistical parameters, such as the probability value (*p* value), the multiple correlation coefficient (*R*^2^), and adjusted multiple correlation coefficient (adjusted *R*^2^). Contour plots for control factors versus response factors were also presented using the software. To determine the optimized process parameters, the empirical models, which interpreted the relationship between control and response factors, were used. The target response values were the following: maximum tablet tensile strength within the lower (≥ 125 N/cm^2^) limit; minimum Carr’s index within the upper (≤ 15) limit; optimal intermediate ranges of dissolved drug at 5 min (72.29–83.29%), 10 min (78.68–88.58%), 15 min (85.00–92.10%), and 30 min (87.21–94.47%). In addition, 95% confidence intervals for the optimal condition were used for the control strategy.

To prepare the test tablet, telmisartan granules were generated by high-shear wet granulation, and Table 1 presents the granule components. Solving agents were sodium hydroxide and meglumine. PVP K25 was used as the binding agent. The telmisartan suspension was prepared by the following: i) dissolving solving agents in 50% (*v*/*v*) ethanol at ambient temperature, ii) introducing telmisartan and binding agent into the i) solution at ambient temperature, and stirring at 50 rpm until it became transparent, iii) the suspension was then stored until use at ambient temperature. To remove any aggregates, F-MELT^®^, D-mannitol dicalcium phosphate, crospovidone, and MCC were filtered with a #30-mesh sieve and then blended for 3 min at 200 rpm; the powder mixture from each experiment was 475 g. A high-shear granulator (Mixer-granulator P 1, DIOSNA Dierks & Söhne GmbH, Germany) with a capacity of 1.23 L was used to prepare the granulation. The agitator speed, massing time, and binding suspension addition rate were set to the predetermined values of the experimental design, and the chopper speed was 2500 rpm. The end point of granulation was determined by monitoring agitator torque. The agitator torque profile provides wet granulation process stages, such as stages 1–4 [40]. Stage 1 represents the initiation of wetting and hence the agitator torque remains relatively constant. Stage 2 represents that agglomeration begins and agitator torque increases. Stage 3 involves the generation of useful granules and a plateau in agitator torque. Stage 4 corresponds to over-wetting with fluctuations in agitator torque. The granules were dried in an oven at 60 °C for 1 h. IQAs, such as granule hardness, Carr’s index, granule true density, granule bulk density, granule FE, and granule particle size were evaluated using the dried granules. The dried granules were introduced into a stainless steel double cone blender (Kopamtec, Anyang, Korea) with a capacity of 20,000 cm^3^ and a fill-weight of approximately 30% of the mixer volume. Magnesium stearate was introduced into the blender and then blended at 50 rpm for 10 min. The resulting granules were compressed on a single-punch hydraulic laboratory press (Ichihashi Seiki Co., Ltd., Kyoto, Japan) at 15 MPa using a rectangle-shaped punch (15 × 8 mm). CQAs were evaluated using the tablets. 

### 2.4. Measurement of Quality Attributes

#### 2.4.1. Measurement of Granule Hardness and Tablet Tensile Strength

The granule strength was measured with a texture analyzer (TA.XT plusC from Stable MicroSystems, Godalming, UK), which has a 500 N force capacity, 0.1 g force resolution, and is equipped with a 50 kg·f loadcell. The dry granules were individually compressed with a 10 mm steel probe. The trigger force was set as 0.001 N. The force versus distance were recorded to calculate the total work (area under the curve). More than 30 granules were evaluated to obtain statistically reliable results. The means of 30 determinations were used to calculate the granule strength for each experiment.

The tablet tensile strength was evaluated by compressing diametrically using a texture analyzer (TA.XT plusC, Stable Micro Systems Ltd., Godalming, UK), and tensile strength was calculated using Fall and Newton’s method [41] as shown in Equation (1):

(1) $$Tensile\;strength=\frac{F}{\pi RT}$$

where *F* is the force required to break the tablet (N), *R* is the radial length of the tablet (mm), and *T* is the thickness of the tablet (mm). The tablet was compressed with a 20 mm steel probe. The trigger force was set as 0.001 N. The force versus distance were recorded to calculate the maximum force. The means of five determinations were used to calculate the tablet tensile strength for each experiment. 

#### 2.4.2. Measurement of Granule Density and Carr’s Index 

An MT-1000 instrument (Seishin Enterprise Co., Tokyo, Japan) was used to evaluate the bulk and tap densities of the granules. To determine the bulk density, the granules were passed through a #18 sieve to break up agglomerates that might have formed during storage. An excess of granules was introduced into a 100 mL cylinder until it overflowed and then the top was carefully scraped to remove the excess granules from the top of the cylinder. The granule weight was accurately measured. The tapped density was determined with a 100 mL cylinder. The granules were introduced into the cylinder and then the cylinder was tapped with 250 drops per min with a fixed drop of 14 ± 2 mm. Each analysis was repeated three times. Carr’s index was determined using Equation (2).

(2) $$\mathrm{Car}\;r\;{}^{\prime }\;s\;\mathrm{Index}=\frac{\mathrm{tapped}\;\mathrm{density}-\mathrm{bulk}\;\mathrm{density}}{\mathrm{tapped}\;\mathrm{density}}\times 100\%$$

The means of three determinations was used to calculate the granule density and Carr’s index for each experiment.

The true density of the granules was evaluated with a helium pycnometer (AccuPyc 1330; Micromeritics Instrument Co., Norcross, GA, USA). Before measurement, the instrument calibration was performed with a standard steel ball to increase the accuracy of the pycnometer. The granule weight was accurately measured, and it was introduced into the sample cell. The sample cell was filled with helium gas and the gas was discharged into a second empty cell. The granule volume was calculated by measuring pressure by filling the sample cell with helium gas. The means of three determinations were used to calculate the granule true density for each experiment.

#### 2.4.3. Measurement of Particle Size 

The laser diffraction method was used to evaluate the particle size distribution of the granules. Approximately, a 5 g granule size was introduced into a HELOS particle size analyzer (Clausthal-Zellerfeld, Germany) ranging from 0.1 µm to 8750 µm. The particle size distribution diameters (*d*_10_, *d*_50_, and *d*_90_) were determined. The measurements were repeated three times and the mean value of *d*_50_ was used as particle size in this study. 

#### 2.4.4. Powder Property Analysis Using a Rheometer

A Freeman FT4 powder rheometer (Freeman Technology, Micrometitics Instrument Corporation, Norcross UK) was used to analyze granule flow energy (FE). A conditioning cycle was completed prior to every test to remove the variability introduced by the operator during loading of the sample and any residual compaction from previous tests. A blade (stainless steel blade of diameter D_b_ = 23.5 mm) moved downward into and through a powder bed contained within a cylindrical vessel with an inside diameter D_v_ = 25 mm and volume of 25 mL. The blade speed was 100 mm/s with a −10° helix angle. As the blade moved through the sample, the FT4 measured both rotational and vertical resistances, in the form of torque and force, respectively. It was the composite of these two values that quantified the granule FE. It was calculated with the Equation (3):

(3) $$\mathrm{granule}\;\mathrm{FE}=\int_{0}^{L}\left(\frac{T}{R\tan\alpha }+\;F_{base}\right)dH$$

where *R* is the agitator blade radius, *T* is the torque acting on the agitator blade, *α* is the helix angle, and *L* is the penetration depth. 

#### 2.4.5. In Vitro Dissolution Test

According to the USP Apparatus 2 guidelines (paddle method), the in vitro dissolution test was conducted (DT 720, ERWEKA GmbH, Germany) with 900 mL of dissolution buffer (50 mM phosphate buffer, pH 7.5) maintained at 37 ± 0.5 °C. The paddle speed was maintained with 75 rpm. At predetermined time intervals, sample aliquots (5 mL) were withdrawn with 15 mL syringes. These were filtered through a 0.45 µm membrane syringe filter. Quantitative analysis for drug content was performed using an HPLC system (Agilent Technologies, Santa Clara, CA, USA) with UV detection at a wavelength of 237 nm. A Supelco Discovery C18 column (4.6 × 250 mm, 5 µM) (Sigma-Aldrich, St. Louis, MO, USA) was used and maintained at 40 °C. The mobile phase was a 45:55 volume mixture of aqueous buffer (pH 2.8, 20 mM phosphate buffer prepared with ammonium dihydrogen phosphate and phosphoric acid) and methanol. The injection volume was 20 µL and the flow rate was 1.0 mL/min. The cumulative percentage of the released drug was calculated, and the results are presented as the mean value of at four tablets for each experiment. 

### 2.5. Multivariate Analysis between IQAs, CQAs, and CPPs 

The combination of experimental design and MVA has grown to be a more powerful system. The commonly used methods include PCA and PLS. PCA is a statistical method that visualizes the relationship between independent variables (X) by shortening multidimensional data to be analyzed into two or three dimensional data. The PLS is a statistical method for establishing the relationship between the independent variable (X) and the dependent variable (Y) based on the principle of PCA described above. That is, by applying PCA to each of the X and Y variables, T and U, consisting of the score values of X and Y, are projected into a new space to obtain a linear regression model [2,3,4,42,43].

In this study, PCA and PLS were performed with SIMCA^©^ software (Sartorius Stedim Biotech., version 15, Umeå, Sweden) to evaluate the mutual relation between both IQAs of processes and drug product CQAs, and variable relationships in the experimental design of process parameters for high-shear wet granulation to prepare telmisartan granules. Moreover, the best fitted regression models between IQAs and CQAs were constructed with a prediction interval (95% PI), a confidence interval (95% CI), and a *p* value to be applicable at the pilot scale. 

## 3. Results and Discussion

### 3.1. Risk Assessment 

A risk assessment of the manufacturing process was performed to identify the high risk process parameters that might have significant effects on the CQAs of the drug product. The IQAs of the output material from the manufacturing process steps that impacted the final drug product CQAs were simultaneously identified. The quality target product profile (QTPP) was defined with the analysis of the control tablet and pharmacopeia [33]. The dosage form, dosage design, route of administration, and stability were based on the analysis of the control tablet because generic drug products are asked to have the same or better quality for pharmaceutical equivalence requirements. Therefore, the above product profiles were not established as CQAs in this study. 

Generally, the IQAs (Carr’s index, granule size, granule hardness, granule true density, granule bulk density, and granule FE) could potentially be influenced by the process parameters and these might be significantly related to the CQAs. Carr’s index could indicate the flowability of the granule. A poorly flowable granule has a Carr index greater than 25, whereas a Carr’s index below 15 is considered to be an indication of good flowability [44]. It is calculated with bulk density and tapped density, which could be significantly influenced by the process parameters [8,19,34,45]. Based on our experience related with the development of similar processes, the particle size distribution of granules and granule hardness manufactured by high-shear wet granulation could significantly influence drug product quality, such as dissolution, tablet tensile strength, content uniformity, and bioavailability [8,19,46,47]. However, the target values of the IQAs, except Carr’s index, could not be determined before the development of the mutual effect with the CQAs. Therefore, the target values were not defined in the risk assessment at early development stages. 

The CQAs (friability, tablet tensile strength, content uniformity (CU), assay, and dissolution) could potentially be influenced by process parameters. Friability might be critically related to the safety and efficacy of the drug product. More than 1.0% (*w*/*w*) of weight loss can cause a significant impact on patient safety and efficacy [33]. However, friability of the test tablet is controlled by stricter standards as seen in the target value; minimized to less than 0.25% (*w*/*w*). Tablet tensile strength could affect the dissolution profile, pharmacokinetic parameters, and disintegration, which is directly related to the safety and efficacy of the drug product. A strong or weak tablet indicates excessive bonding between API and excipients, which can lead to an improper dissolution profile. The bonding could be influenced by process parameters. Variability in content uniformity or assay is related to the safety and efficacy of the drug product. Process variables could affect the assay or content uniformity of the drug product. The dissolution profile could be directly related to the safety and efficacy of the drug product, which significantly influences bioavailability. Therefore, the above mentioned the IQAs and CQAs should be evaluated, and the mutual interactions between the IQAs and CQAs were analyzed in detail during process parameter development studies. 

**Figure 1:**
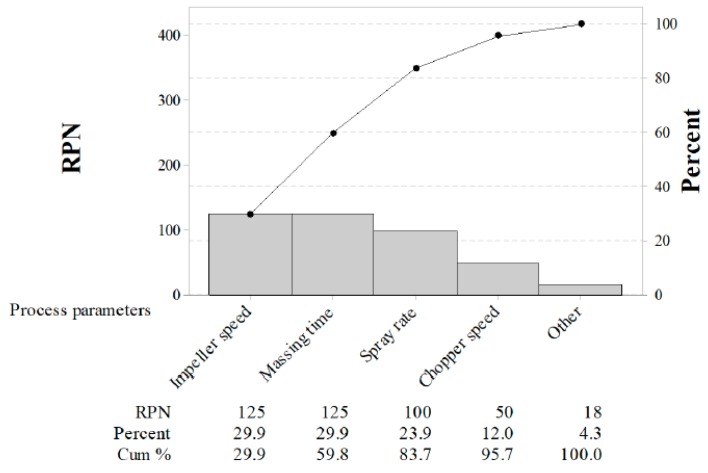
Pareto chart of the risk priority number (RPN). Individual values are represented in descending order by bars, and the cumulative total is represented by the line. The left vertical axis is the RPN. The right vertical axis is the cumulative percentage of the RPN.

As shown in Table 2, FMEA was performed to identify the effects of CPPs on CQAs and IQAs. Previous experience with these process steps and information about the control tablet from the published literature [8,9,14,19,40,41,48] or prior analysis were used to determine the degree of risk associated with process parameters and its potential to impact the CQAs and IQAs. For the risk assessment, three categories (high, medium, and low) were mapped based on the degree of RPN. Moreover, a pareto chart was constructed with RPN as shown in Figure 1. The RPN and the cumulative total number were presented in descending order by the bars and line, respectively. The RPN of agitator speed, massing time, and binding suspension addition rate were 125, 125, and 100, respectively. Moreover, the cumulative percent of the three process parameters was 83.7%. Based on the risk assessment, the agitator speed, binding suspension addition rate, and massing time were identified as potentially having a risk impact on CQAs and IQAs and these were evaluated in detail with experimental design. 

### 3.2. Effect of High-Shear Wet Granulation Process Parameters on IQAs and CQAs

A response surface design was used to evaluate the effect of the three control factors on the three response factors. Table 3 presents the experimental matrix for the 15 experiments and experiment results. The mathematical empirical models were generated with coded values for factor levels. A positive coefficient indicated a synergistic effect on the response factor, whereas an antagonistic effect on the response factor was a negative coefficient. In addition, a larger coefficient indicated a stronger effect of the independent variable of the response [49,50,51]. 

However, CQAs, such as friability, content uniformity, and assay, were excluded to determine operating space because all run orders demonstrated acceptable values, such as content uniformity (ranging from 1.2–2.5% RSD), friability (<0.2% weight loss when compressed using 15 MPa of force), and assay (ranging from 98.6–100.5%). Moreover, none of the process parameters showed a significant impact on these CQAs. 

#### 3.2.1. Significant Factors for Granules Hardness (*q*_1_)

Granule hardness is one of the most important parameters that can affect the disintegration time, tensile strength, and dissolution rate of tablets. Moreover, granules should have sufficient strength to resist compression. For this reason, granule hardness was evaluated in this study with a texture analyzer. As shown in Table 3, the granule strength manufactured by different process parameters ranged from 0.90–5.00 N. The effect of process parameters on granule hardness was presented as the empirical model described by Equation (4).

(4) $$q_{1}=2.16+1.49p_{1}-0.64p_{2}+O.29p_{3}+0.55p_{1}{}^{2}$$

**Figure 2:**
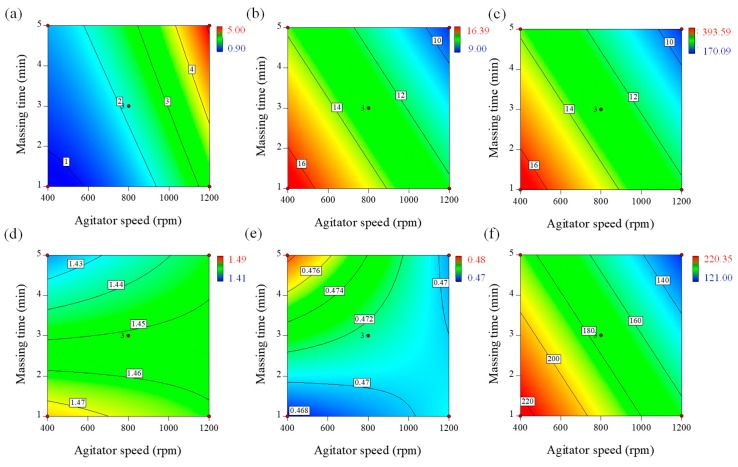
Contour plots correlating response factors (IQAs) to optimize process parameters of high-shear wet granulation for the telmisartan test tablet. (**a**) Granule hardness, (**b**) Carr’s index, (**c**) Granule size, (**d**) True density, (**e**) Bulk density, and (**f**) Granule flow energy (FE).

All of the factors had significant effects on granule hardness (*p* < 0.05). Agitator speed (*p*_1_) and massing time (*p*_2_) had positive effects, whereas binding suspension addition rate (*p*_3_) had a negative effect on granule hardness. This is presented in the contour plot in Figure 2a. The plot shows that higher agitator speeds and longer wet massing times increased granule hardness; granule hardness was also increased at low water addition rates. Higher agitator speeds and longer massing times could increase granule density and hardness because high shearing forces consistently exist between the particles [8,13,14,52]. Thereore, the bonding strength between particles strengthens the resistance to the separating forces. 

#### 3.2.2. Significant Factors for Carr’s Index (*q*_2_)

The flowability of the granules is significantly influenced by the granulation process [53]. Carr’s index is frequently used in pharmaceutics as an indication of the flowability of a powder. In high flowability granules, the bulk density and tapped density would be close in value; therefore, Carr’s index would be small. In contrast, in poor flowability powders with greater interparticle interactions, the difference between the bulk and tapped densities would be larger; therefore, Carr’s index would be larger. Carr’s index greater than 25 is considered an indication of poor flowability, whereas below 15 indicates good flowability [17,33]. As shown in Table 3, the Carr’s indexes manufactured by different process parameters ranged from 9.00–16.39, which could be interpreted as an indication of good flowability [54]. Regression analysis of the response factor, with significance at *p* < 0.05, was used to generate the empirical model described by Equation (5).

(5) $$q_{2}=13.05-2.25p_{1}-1.45p_{2}$$

Agitator speed (*p*_1_) and massing time (*p*_2_) had significantly negative effects on granule flowability. The effect of agitator speed and massing time on Carr’s index of the granules is presented in the contour plot in Figure 2b. An increase in agitator speed and massing time could generate high shearing forces between the particles. Consequently, the growth and densification of the granules would progress. Therefore, Carr’s index decreases with increased agitator speed and massing time.

#### 3.2.3. Significant Factors for Granule Size (*q*_3_)

The process parameters in the high-shear wet granulation process might significantly influence granule size. Granule size distribution might be significantly related to desired qualities in key areas, such as product uniformity, dissolution, flow, hardness, and bioavailability. Granule size is also used to optimize downstream processing as it relates to blending and compression. As shown in Table 3, the granule sizes manufactured by different process parameters ranged from 170.09–393.59 µm, representing the surface mean diameter. Regression analysis of the response factor, with a significance value of *p* < 0.05, was used to generate the empirical model described by Equation (6).

(6) $$q_{3}\;=264.61+87.66p_{1}+19.05p_{2}$$

Equation (6) and the contour plot in Figure 2c show that the agitator speed (*p*_1_) had the largest effect on the surface mean diameter, followed by the massing time (*p*_2_). Both variables have a synergistic effect on the surface mean diameter. Long wet massing times and high agitator speeds may result in granule growth by coalescence, but large granules may undergo breakage until a steady state of granule size is reached. This implies that agitator speed and massing time have greater potential for controlling the granulation process [19,55,56].

#### 3.2.4. Significant Factors for Granule True Density (*q*_4_) and Bulk Density (*q*_5_)

The granule true density after high-shear wet granulation was determined using a pycnometer. As shown in Table 3, the granule true density manufactured by different process parameters ranged from 1.409–1.491 g/cm^3^. Regression analysis of the response factor, with a significance value of *p* < 0.05, was used to generate the empirical model described by Equation (7).

(7) $$q_{4}\;=1.45+0.0025p_{1}-0.0173p_{2}-0.0024p_{3}+0.0092p_{1}p_{2}+0.0285p_{2}p_{3}$$

The equation shows that the significant factors affecting granule true density were agitator speed (*p*_1_), binding suspension addition rate (*p*_3_), massing time (*p*_2_), the mutual interactions between agitator speed and massing time, and mutual interactions between binding suspension, addition rate, and massing time. The contour plot presented in Figure 2d shows the effect of binding suspension addition rate and massing time on granule true density. It is evident that the granule true density increased with decreasing binding suspension addition rate and massing time (negative effect). Meanwhile, these two parameters exhibited a strong positive interaction (i.e., massing time showed a larger impact on the granule true density when using a larger binding suspension addition rate).

As shown in Table 1, the granule bulk density manufactured by different process parameters ranged from 0.467–0.479 g/mL. Regression analysis of the response factor, with a significance value of *p* < 0.05, was used to generate the empirical model described by Equation (8).

(8) $$q_{5}\;=0.4715+0.0015p_{1}+0.0024p_{2}+0.002p_{3}-0.0030p_{1}p_{2}+0.0034p_{2}p_{3}$$

The equation shows that agitator speed (*p*_1_), binding suspension addition rate (*p*_3_), massing time (*p*_2_), the mutual interactions between them were the significant factors affecting granule bulk density. The effect of binding suspension addition rate and massing time on the granule bulk density is presented in Figure 2e as a contour plot. The granule bulk density increased with increasing binding suspension addition rate and massing time (positive effect). These two parameters also exhibited a strong positive interaction. Meanwhile, the mutual interaction between agitator speed and massing time had a negative effect on the granule bulk density. 

#### 3.2.5. Significant Factors for Granule FE (*q*_6_) 

Recently, the flow properties of pharmaceutical powders have been characterized with the rheological measurements [57,58,59,60]. As the blade passed through a predetermined distance into a powder bed, the torque and force on the blade were measured [60,61]. As shown in Table 3, the granule FE values are ranged from 121–220. Regression analysis of the response factor, with *p* < 0.05, was used to generate the empirical model described by Equation (9).

(9) $$q_{6}=175.44-30.22p_{1}-19.54\;p_{2}+3.58p_{3}+8.97p_{2}p_{3}$$

The equation shows that the significant factors affecting granule FE were agitator speed (*p*_1_), massing time (*p*_2_), binding suspension addition rate (*p*_3_), and the mutual interactions between binding suspension addition rate and massing time. The contour plot presented in Figure 2f shows the effect of blade speed and massing time on the granule FE. Based on the coefficients of the empirical model, the granule flow energy decreased with increasing blade speed and massing time (negative effect). 

#### 3.2.6. Significant Factors for Tablet Tensile Strength (*q*_7_)

Tablet dosage forms should have a certain strength to resist the mechanical stress of handling during manufacture, packaging, and shipping [31]. In addition, previous studies reported that tablet strength was related to tablet disintegration time, dissolution profiles, and bioavailability [62]. Table 3 shows that the tablet tensile strengths of different formulations ranged from 125.88–145.51 N/cm^2^. Regression analysis of the response factor, with a significance of *p* < 0.05, was used to generate the empirical model described by Equation (10).

(10) $$q_{7}\;=137.54+6.13p_{1}+3.25p_{2}-0.70p_{3}-1.59p_{1}p_{2}-1.56p_{1}p_{3}$$

**Figure 3:**
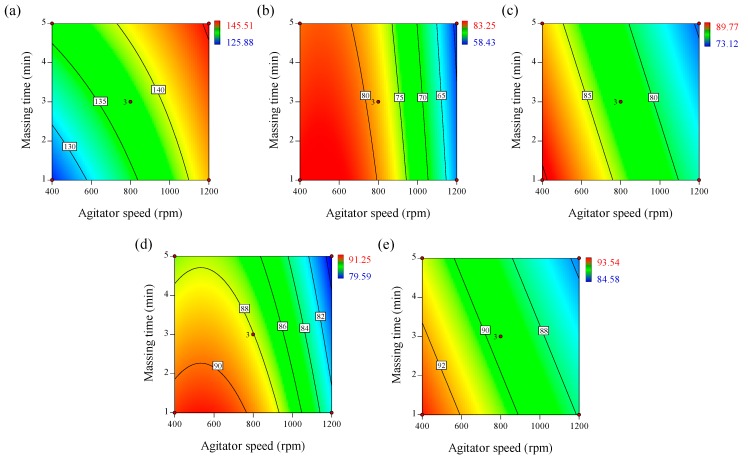
Contour plots correlating response factors (CQAs) to optimize process parameters of high-shear wet granulation for the telmisartan test tablet. (**a**) Tensile strength, (**b**) % dissolution at 5 min, (**c**) % dissolution at 10 min, (**d**) % dissolution at 15 min, and (**e**) % dissolution at 30 min.

This equation and the contour plot in Figure 3a show that agitator speed (*p*_1_) had the highest effect on tablet tensile strength, followed by massing time (*p*_2_). The positive coefficients of agitator speed and massing time indicate that higher values for these parameters result in higher tensile strength. binding suspension addition rate (*p*_3_) had a negative effect on tensile strength. The mutual interactions between the agitator speed and binding suspension addition rate, and between the agitator speed and massing time had negative effects on the tensile strength of the tablet. 

#### 3.2.7. Significant Factors for Dissolution (*q*_8_–*q*_11_)

Table 3 shows the dissolution results of the telmisartan tablets manufactured with different process parameters. Regression analysis of the response factors, with a significance value of *p* values < 0.05, was used to generate the empirical models described by Equations (11)–(14).

(11) $$q_{8}=78.45-10.86p_{1}-1.35p_{2}-7.24p_{1}^{2}$$

(12) $$q_{9}=82.51-5.97p_{1}-1.89p_{2}$$

(13) $$q_{10}=88.04-4.07p_{1}-1.64p_{2}-3.05p_{1}^{2}$$

(14) $$q_{11}=89.50-2.69p_{1}-1.10p_{2}$$

As shown in Equations (11)–(14) and the contour plots in Figure 3b–e, the agitator speed (*p*_1_) and massing time (*p*_2_) could have significant, negative effects on dissolution, whereas the binding suspension addition rate (*p*_3_) may have no effect on dissolution. From 5–30 min, the larger *p*_1_ value could indicate that dissolution over the initial 30 min is significantly controlled by agitator speed. The blade presents the mechanical energy to generate granules, whereas the mechanical energy generated by the chopper breaks up granules that are generated. Thus, the response of the granules, such as granule size and growth rate could be significantly influenced by the energy resulting from the agitator and chopper speed [63,64]. Generally, larger granules have a lower dissolution profile than smaller granules because smaller granules have a larger surface area in contact with the dissolution medium. Therefore, increased agitator speed could result in increased granule size, negatively influencing the dissolution profile. The massing time also had a negative effect on the dissolution profile. A high massing time could present the mechanical energy required for mixing the feed powder. This mechanical energy could increase the granule size; thus, the dissolution profile could be negatively influenced by the massing time. 

### 3.3. Optimal Process Parameters and Monte Carlo Simulations

**Figure 4:**
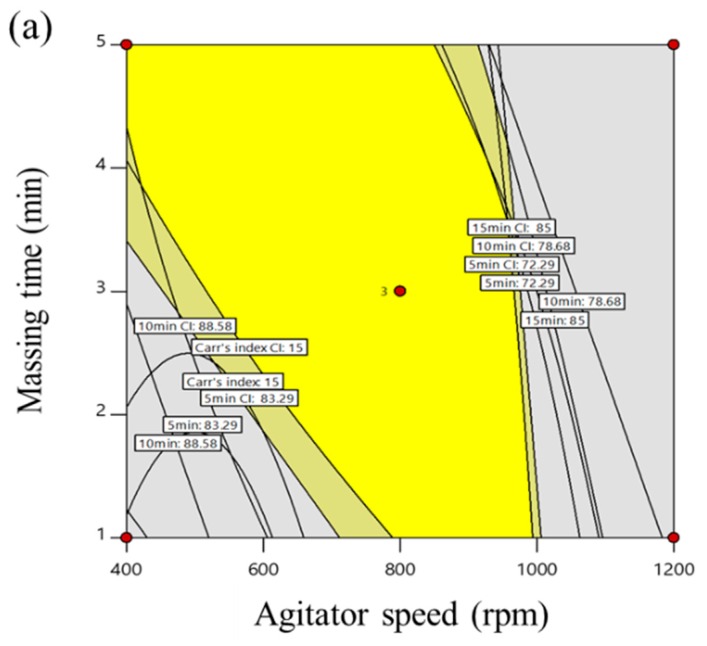

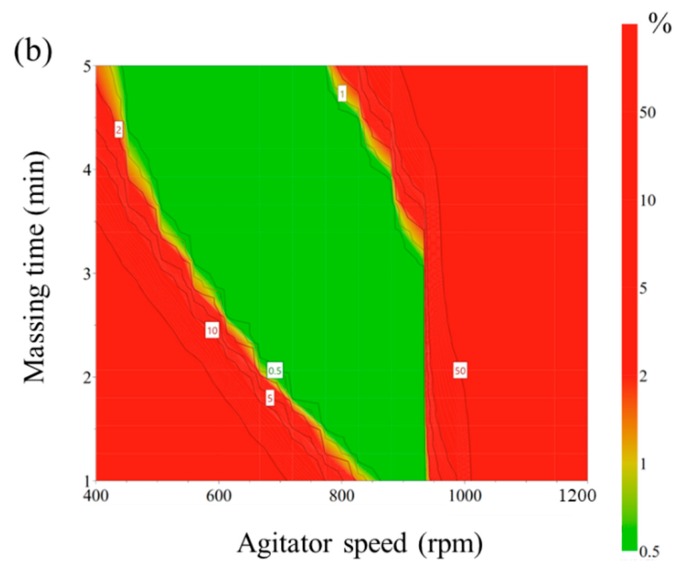
(**a**) Operating spaces (overlay plot) to optimize process parameters of high-shear wet granulation for the telmisartan test tablet. The yellow area is the operating space with 95% confidence intervals. (**b**) Monte Carlo simulation results for the operating space. The green area shows a quality certainty of 99.9.

The optimization of the process parameters was conducted with statistically validated empirical equations, which were used to interpret the relationship between the control and response factors. The optimization condition for the high-shear granulation process presented the following: maximum tablet tensile strength within the lower (≥ 125 N/cm^2^) limit; minimum Carr’s index within the upper (≤ 15) limit; optimal intermediate ranges of dissolved drug at 5 min (72.29–83.29%), 10 min (78.68–88.58%), 15 min (85.00–92.10%), and 30 min (87.21–94.47%). In addition, a 95% confidence interval of the optimal condition was used for the control strategy. Based on these conditions, the responses were then combined to reveal the overall optimum region. This optimum region of the high-shear granulation process is shown in Figure 4a, in which the yellow area indicates the values of the control factors that achieve the desired responses simultaneously. To investigate the robustness of the optimum region, Monte Carlo simulations were performed with MODDE^®^ software (Sartorius Stedim Biotech., version 12.0.1, Umeå, Sweden). To find the most robust process parameters in the operating space, the setting point should have a DPMO (Defects Per Million Opportunities) around or less than the specification. A DMPO result of 1000 means 0.1% risk of failure. The result is presented in Figure 4b with the probability of failure. The green area shows a quality certainty of 99.9%. Furthermore, as the probability of failure increased, the area was represented with red color. This suggested that it was not safe to obtain the target response at a higher agitator speed and higher massing time. However, agitator speeds ranging 700–900 rpm and massing times ranging 2.5–3.5 min in high-shear wet granulation had a low probability of failure less than 1%. To validate the optimal process parameters, these settings were performed at the lab scale. The spray rate was fixed with 5.20 mL/min. The agitator speed was selected 700, 800, and 900 rpm in the operating space. The massing time was also selected in the operating space as 2.5, 3.0, 3.5 min. Table 4 presents the experimental results with the target values of the response factors. Additionally, differences in biases and relative bias percentages between the response values generated by the optimal process parameters and the target values were small. The difference in biases relative bias percentages were lower than 2.20 and 5.08. This suggests that a risk reduction for the high-shear wet granulation at the lab scale can be obtained using QbD approach that controlled the development process from the initial stage with RA to robust operating space. 

### 3.4. Multivariate Analysis between CQAs of Drug Product and IQAs of Process 

**Figure 5:**
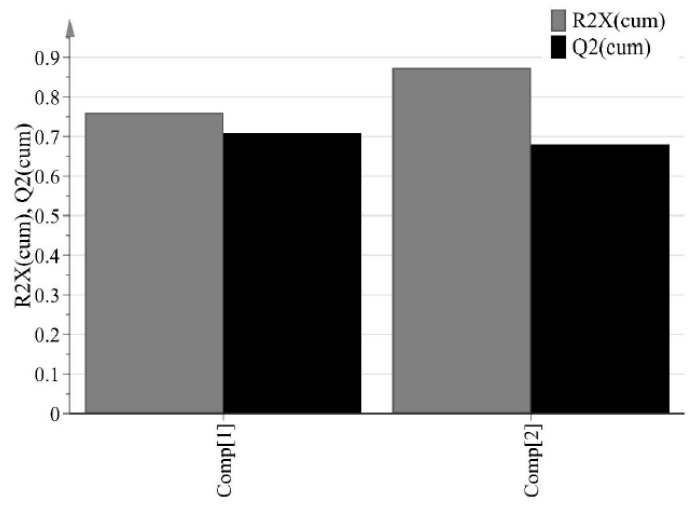
Cumulative R2 (R2X(cum)) and Q2 (Q2(cum)) of response factors (IQAs and CQAs) for two principal components.

**Figure 6:**
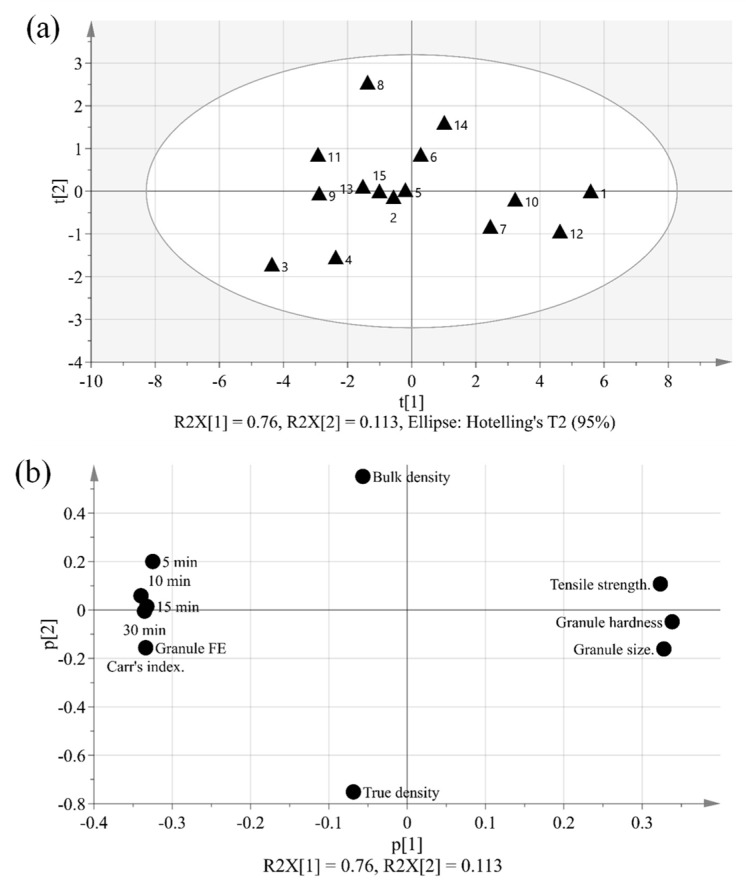
(**a**) Score scatter plot with two principal components (t[1] and t[2]) and (**b**) loading plot (p[1] and p[2]) presenting variable relationships.

**Figure 7:**
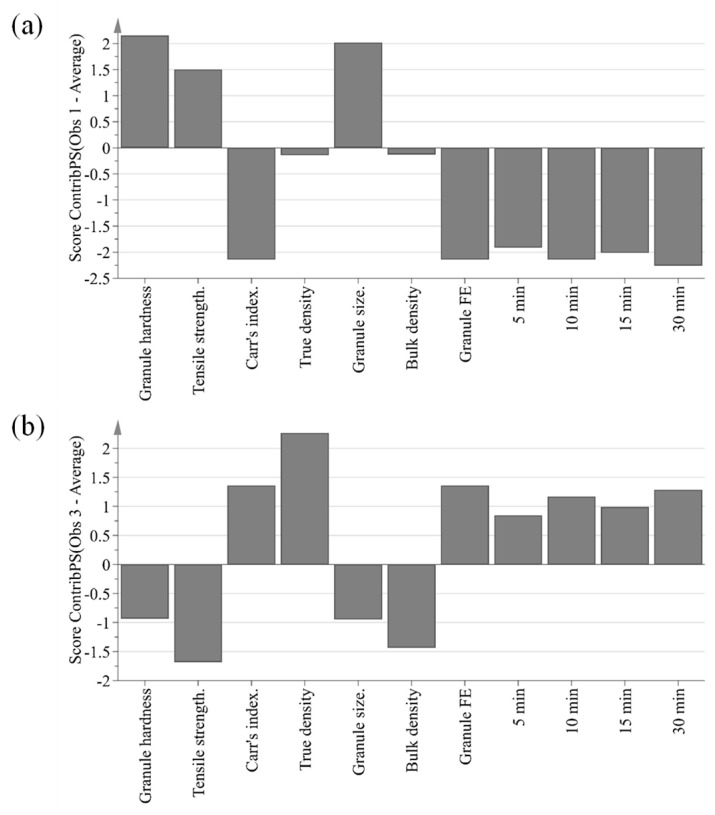
Score contribution plots showing variables contributing to the difference between run order 1 (**a**) or run order 3 (**b**) and the average of all run orders.

The purpose of PCA is to identify relationships between IQAs and CQAs in the experimental design. All 15 experimental design samples were included in the PCA. 11 variables were analyzed, including IQAs, such as granule size, granule hardness, Carr’s index, granule FE, bulk density, and true density, and CQAs, such as tensile strength and % dissolution at each time point. Two PC models were fitted to the variables. As shown in Figure 5, the first and second PCs (P[1] and P[2], respectively) explained 76.0% and 11.3% of the overall variability in the dataset, respectively. R2 is used as a measure of how well the variance of the data is explained in the model, and Q2 is used as a measure of how well the variance of the data is predicted in the model. The closer each value is to 1.0, the better the fit of the model. A score scatter plot in Figure 6a suggests there are no groups and no outliers outside the ellipse (95% confidence interval). In addition, the center point samples (run order 2, 5, and 15) were located near each center, indicating good reproducibility. Run order 1 and 3 were located further away from the other run orders. Score contribution plots of run order 1 and 3 are shown in Figure 7. It identifies why these differ more and which variables are the greatest contributing factors to the differences. The variables with larger positive and lesser negative values had significant effects on differentiating run order 1 and 3. The variables were shown to have completely opposite effects. This suggests that agitator speeds ranging 400–1200 rpm and massing time ranging 1–5 min in high-shear wet granulation have significant effects on IQAs and CQAs.

The dataset can be displayed on a loading plot. The loading value reflects the weight of the variable relative to the principal component. In the score and loading plot, if the variable is highly correlated with the principal component, a positive loading value or a negative loading value is assigned. The range is −1 to +1. If the value is near 0, it indicates that the variable does not provide information to this main component. The loading plot can be used to identify the covariance between variables and to interpret the pattern observed in the score plot. As shown in Figure 6b, the loading scatter plot can elucidate the relationship between the IQAs and CQAs. In the case of P[1], which fully explains the degree of variance in the overall data, tensile strength (CQA), with a strong positive loading value, and Carr’s index and granule Fe, with strong negative loading values, can be regarded as having a negative relationship, because they are located on opposite sides. In addition, it can be seen that granule size and granule hardness, with strong positive loading values for P[1], and are located on the opposite side of % dissolution with a strong negative loading value; thus, they also have a negative relationship.

**Figure 8:**
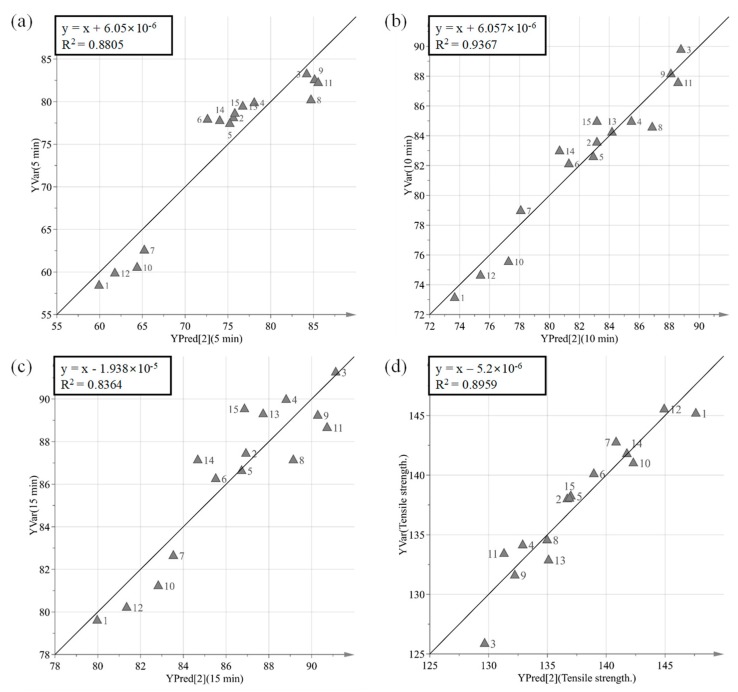
Predicted versus measured plots for tensile strengths (CQAs). (**a**) % dissolution at 5 min, (**b**) % dissolution at 10 min, (**c**) % dissolution at 15 min, and (**d**) tensile strength.

**Figure 9:**
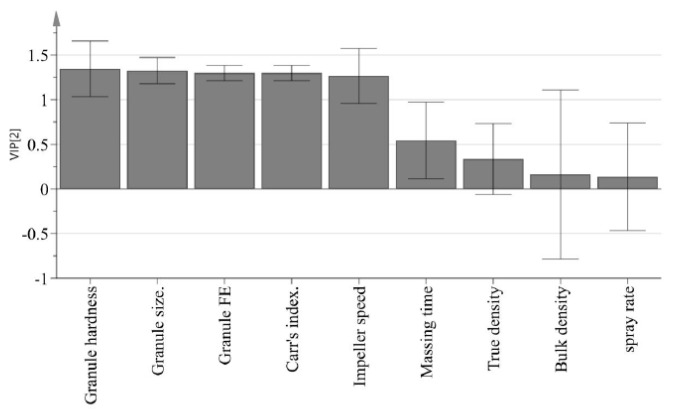
VIP (variable importance) plot presenting very important variables in terms of CQAs.

The purpose of PLS is to identify how all available process parameters and IQAs impact CQAs. PLS was used to define the relationship between X variables (process parameters and IQAs) and Y variables (CQAs). Figure 8 presents a predicted versus observed plot for CQAs from calibration models, which is presented by a solid black line. The R2 had % dissolution at 5 min, 10 min, and 15 min and tensile strength at 0.8805, 0.9367, 0.8364 and 0.8959, respectively. This might indicate a good model fit. As shown in Figure 9, the VIP (variable importance) presents the importance variables in the model both with respect to Y (CQAs) and X (process parameters and IQAs). VIP is normalized and the average squared VIP value is 1. Variables in the model with a VIP >1 are important to the model [1]. It appears that granule hardness, granule size, granule FE, Carr’s index, and agitator speed were important variables with respect to CQAs. Furthermore, massing time, true density, bulk density, and binding suspension addition rate did not have a significant effect on CQAs.

**Figure 10:**
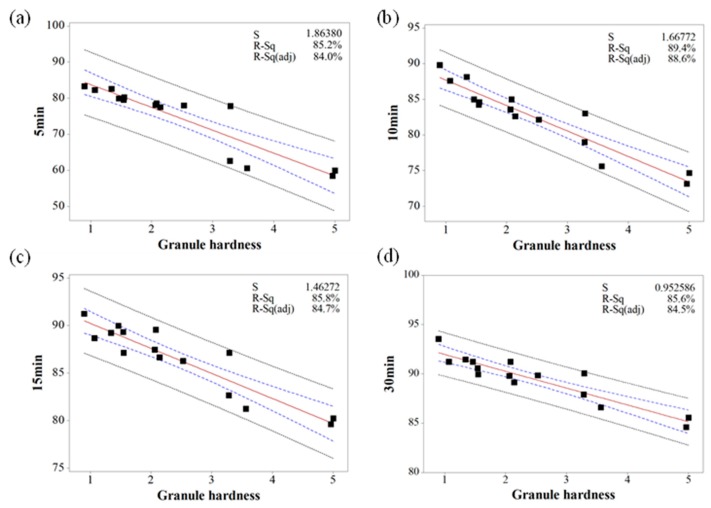
The fitted line plots of % dissolution ((**a**) 5 min, (**b**) 10 min, (**c**) 15 min, and (**d**) 30 min) versus granule hardness. The black dots, red line, blue dotted line, and black dotted line represent the experimental results, best fitted regression, 95% confidence intervals, and 95% prediction intervals, respectively.

**Figure 11:**
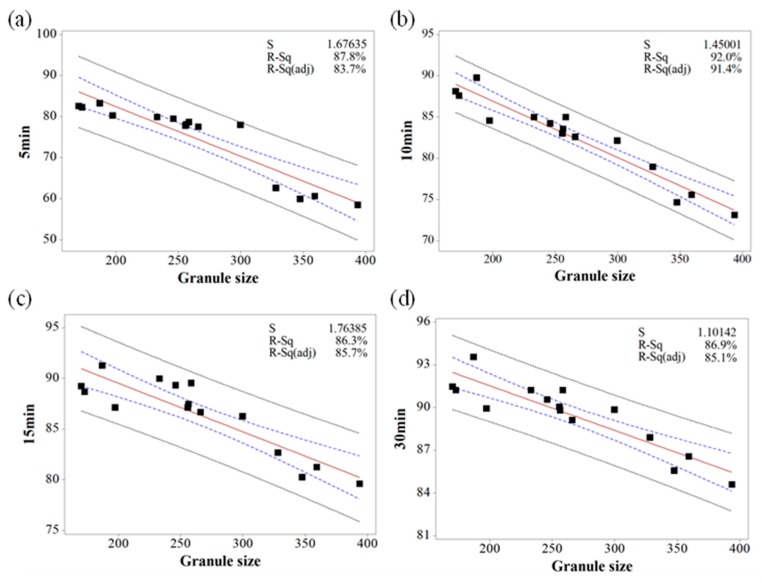
The fitted line plots of % dissolution ((**a**) 5 min, (**b**) 10 min, (**c**) 15 min, and (**d**) 30 min) versus granule size. The black dots, red line, blue dotted line, and black dotted line represent the experimental results, best fitted regression, 95% confidence intervals, and 95% prediction intervals, respectively.

**Figure 12:**
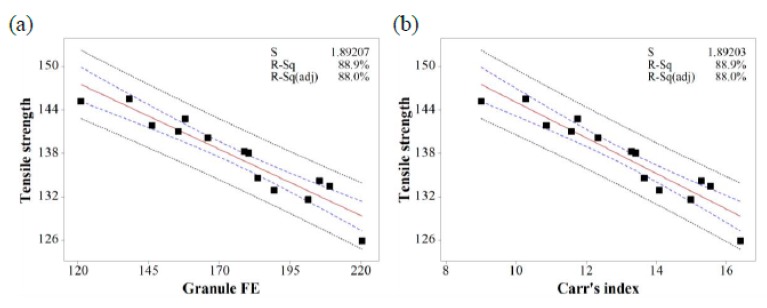
The fitted line plots of (**a**) tensile strength versus granule FE and (**b**) tensile strength versus Carr’s index. The black dots, red line, blue dotted line, and black dotted line represent the experimental results, best fitted regression, 95% confidence intervals, and 95% prediction intervals, respectively.

Based on the MVA, the relationship between IQAs and CQAs (granule size versus % dissolution, granule hardness versus % dissolution, granule FE versus tensile strength, and Carr’s index versus tensile strength) were fitted to establish prediction models with 95% confidence intervals and 95% prediction intervals. Figure 10, Figure 11 and Figure 12 show the fitted line plots. The models contained an adequate number of observations throughout the entire range of all the predictor values. The scatter points generally followed the regression line. There did not appear to be any curvature in the data. The *p* values for the regression models were less than the significance level of 0.05. The S value represents the standard deviation of the distance between the data values and the fitted values. Therefore, the S value can present how well the model describes the response. The lower the value of S, the better the model describes the response. The S values of the prediction models were lower than 3.719, which indicated that the models could accurately predict the response. Moreover, the higher R-Sq (> 80.7%) also indicated a good model fit. Therefore, using these models, CQAs could be accurately predicted with IQAs. In addition, the target values for IQAs could be established with these models.

### 3.5. Scale-Up to Validate the Mutual Effects between CPPs, IQAs, and CQAs

**Figure 13:**
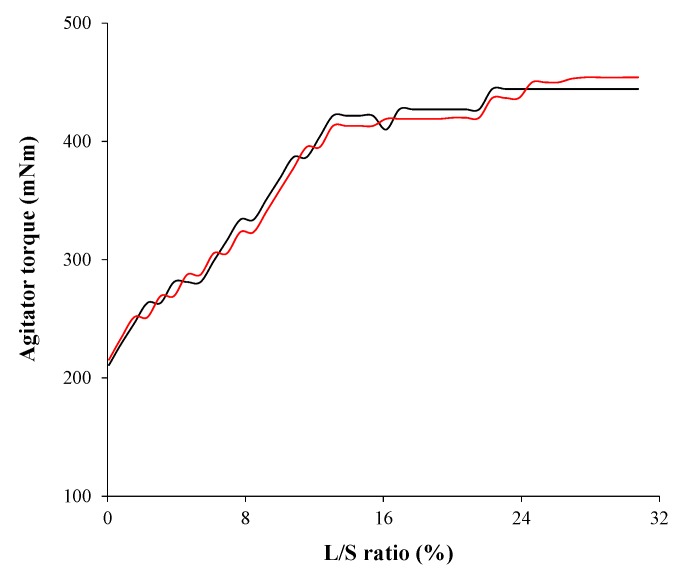
Agitator torque profiles at the lab scale (black line) and pilot scale (red line). The agitator powder consumption is plotted versus liquid/solid (L/S) ratio (%).

Process development using the QbD approach was studied at the lab scale (granulator capacity is 1.0 L). To validate the mutual effects between CPPS, IQAs, and CQAs, and manufacture clinical batch, a scale-up from lab to pilot scale was performed using geometric similarity, agitator torque profile, and agitator blade tip speed. The optimal process obtained from the lab-scale experimental design was used. Based on the experimental design and MVA, IQAs, and CQAs were significantly affected by agitator speed and massing time, whereas binding suspension addition rate did not have a significant effect. Therefore, the agitator blade tip speeds were kept constant regardless of granulator size. The agitator speed used in the scale-up is shown in Table 5. This was calculated from the agitator blade tip speed and the diameters of the agitator blade. Massing time was determined by the agitator torque profile. In various literature, agitator torque profile has been used for a scale-up strategy and granules might have similar properties when agitator torque profiles were similar [30,40,65,66]. The agitator torque profiles at the lab scale and pilot scale are shown in Figure 13. Based on the profiles, it was indicated that granule properties at the lab scale and pilot scale might be similar even with the same mixing time, which was 3 min. A geometric similarity was obtained using a similar granulator (P 6). The Diosna granulator allows bowls of different capacities in the range 1-6 L to be easily interchanged, which means that different batch sizes can be manufactured using the same basic equipment [24,28,29,67]. In addition, the fill levels were maintained at a similar ratio, such as 38.6% at the lab scale and 39.2% at the pilot scale.

Table 6 presents the validated results of IQAs and CQAs at the lab and pilot scale process. The absolute biases and relative bias percentages between the IQAs and CQAs generated by lab and pilot scale process had small differences, which were less than 9.09% (relative biases of friability). The IQA and CQA values might demonstrate similar target values, such as maximum tablet tensile strength within the lower (≥ 135 N/cm^2^) and upper (≤ 145 N/cm^2^) limits; minimum Carr’s index within the lower (≥ 9) and upper (≤ 15) limits; optimal intermediate ranges of dissolved drug at 5 min (72.29–83.29%), 10 min (78.68–88.58%), 15 min (85.00–92.10%), and 30 min (84.47–94.47%); minimum friability less than 0.25%; optimal intermediate ranges of assay (95–105%); minimum content uniformity less than 5.0% RSD. This suggests that the integrated process parameters obtained from experimental design at the lab scale could be applied at the pilot scale with the scale-up strategy. Additionally, the similar relationship between IQAs, CQAs, and CPPs was shown regardless of batch size, it can be used to predict CQAs for control strategy.

## 4. Conclusions

Based on the control strategy, the development of process parameters for high-shear wet granulation was studied at the lab scale with the QbD approach. Risk assessment was conducted to determine the control and response factors and it was based on a primary knowledge and the target values of the control tablet. A response surface design was performed to obtain the optimal process parameters. The predictable empirical equations of best fit were generated with the quantitative effects of the different control factors on the response factors. The operating space at the lab scale was obtained with the empirical equations. The robustness of the operating space was identified with a Monte Carlo simulation. The relationship between IQAs, CQAs, and CPPs was investigated with MVA. The validation results at the pilot scale suggested that the integrated process parameters obtained from experimental design at the lab scale could be applied at the pilot scale with the scale-up strategy. This study suggests that the risk reduction for the manufacturing process could be obtained with integrated process parameters as a result of the QbD approach, and the relationship among IQAs, CQAs, and CPPs could be used to predict CQAs for control strategy and SUPAC (Scale-Up and Post-Approval Guidance).

## Figures and Tables

**Table 1:** Formulation of telmisartan granules.

Purpose	Excipient	Amount (mg/tablet)
Active pharmaceutical ingredient	Telmisartan	80
Solubilizing agent	NaOH	6.7
	Meglumine	24
Binding agent	PVP K25	12
Thicking agent	D-mannitol	166
	MCC 101	39
	Dicalcium phosphate	9.3
Disintegrant agent	F-melt typeC	20
	Crospovidone	118
Lubricant agent	St-Mg	5
Total		480

**Table 2:** Risk assessment based on prior knowledge and experience, and information about the control tablet from published literature or analyses. (RPN: risk priority number; IQAs: intermediate quality attributes; CQA: critical quality attribute; CU: content uniformity; FE: flow energy)

Unit Process	Failure Mode	*S*	*P*	*D*	RPN	Risk Degree	Related IQAs and CQAs
High shear granulation for test tablet	Agitator speed	5	5	5	125	High	dissolution, CU, tensile strength, Friability, granule size, granule hardness, Carr’s index, granule density, granule FE
	Chopper speed	5	2	5	50	Moderate	dissolution, CU, tensile strength, Friability, granule size, granule hardness, Carr’s index, granule density, granule FE
	Solvent spray rate	4	5	5	100	High	dissolution, CU, tensile strength, Friability, granule size, granule hardness, Carr’s index, granule density, granule FE
	Massing time	5	5	5	125	High	dissolution, CU, tensile strength, Friability, granule size, granule hardness, Carr’s index, granule density, granule FE
	Drying temp.	3	1	3	9	Low	tensile strength, Friability, granule hardness, Carr’s index, granule density
	Drying time	3	1	3	9	Low	tensile strength, Friability, granule hardness, Carr’s index, granule density

**Table 3:** Experimental design of high-shear wet granulation process parameters with three input control factors and values of the response factors for different process parameters

Run Order	Control Factors			Response Factors										
	CPP			Intermediate QAs						Drug Product CQAs				
	Agitator Speed (rpm)	Massing Time (min)	Spray Rate (mL/min)	Granule Hardness (N)	Carr’s Index	Granule Size (μm)	Granule True Density (g/cm^3^)	Granule Bulk Density (g/mL)	Granule FE	Tensile Strength (N/cm^2^)	% Dissolution			
											5 min	10 min	15 min	30 min
	*p*_1_	*p*_2_	*p*_3_	*q*_1_	*q*_2_	*q*_3_	*q*_4_	*q*_5_	*q*_6_	*q*_9_	*q*_10_	*q*_11_	*q*_12_	*q*_13_
1	1200	5	5.2	4.96	9.00	393.59	1.438	0.469	121	145.19	83.25	89.77	91.25	93.54
2	800	3	5.2	2.06	13.40	256.00	1.457	0.471	180	138.00	80.21	84.56	87.12	89.94
3	400	1	5.2	0.90	16.39	186.94	1.478	0.468	220	125.88	82.56	88.12	89.22	91.45
4	800	1	3.7	1.46	15.27	232.85	1.492	0.471	205	134.15	82.25	87.58	88.67	91.21
5	800	3	5.2	2.14	13.29	265.95	1.455	0.472	179	138.25	78.11	83.55	87.45	89.79
6	800	5	6.7	2.53	12.33	299.63	1.465	0.479	166	140.12	79.87	84.97	89.97	91.22
7	1200	1	5.2	3.28	11.74	328.04	1.463	0.470	158	142.74	77.45	82.58	86.64	89.12
8	400	5	5.2	1.55	13.65	197.12	1.416	0.479	184	134.55	77.95	82.12	86.25	89.84
9	400	3	3.7	1.34	14.97	170.09	1.456	0.471	201	131.58	79.45	84.21	89.31	90.55
10	1200	3	6.7	3.56	11.56	359.09	1.454	0.473	155	141.00	77.75	82.98	87.12	90.05
11	400	3	6.7	1.07	15.54	172.72	1.443	0.475	209	133.42	78.58	84.97	89.54	91.21
12	1200	3	3.7	5.00	10.27	347.43	1.458	0.468	138	145.51	58.43	73.12	79.59	84.58
13	800	1	6.7	1.54	14.07	245.79	1.434	0.468	189	132.87	62.55	78.95	82.65	87.89
14	800	5	3.7	3.29	10.86	141.80	1.409	0.469	146	255.66	60.54	75.55	81.23	86.57
15	800	3	5.2	2.08	13.40	138.07	1.449	0.471	180	258.23	59.87	74.62	80.22	85.56

**Table 4:** Optimal process parameters, target values, and validated results at the lab scale for the response factors

Optimal Process Parameters			Response Factors					
*x*_1_	*x*_2_	*x*_3_	*q*_1_	*q*_2_	*q*_7_	*q*_8_	*q*_9_	*q*_10_
Agitator Speed (rpm)	Massing Time (min)	Spray RRate (mL/min)	Carr’s index	Tensile Strength (N/cm^2^)	Dissolution at 5 min (%)	Dissolution at 10 min (%)	Dissolution at 15 min (%)	Dissolution at 30 min (%)
700	2.5	5.20	13.87	133.1	79.5	82.39	88.55	90.26
Target values			13.97	135.1	81.05	84.47	89.27	91.45
Absolute viases			0.10	2.00	1.55	2.08	0.72	1.19
Relative biases (%)			4.50	1.47	2.07	2.54	0.82	1.33
700	3.5	5.20	13.12	134.72	79.25	82.23	87.13	87.9
Target values			13.24	136.92	80.37	83.53	88.45	89.89
Absolute viases			0.12	2.20	1.12	1.30	1.32	1.99
Relative biases (%)			5.08	1.61	1.50	1.62	1.54	2.29
900	2.5	5.20	12.79	137.71	75.46	80.72	86.16	87.78
Target values			12.85	138.35	75.62	81.48	87.24	89.10
Absolute viases			0.06	0.64	0.16	0.76	1.08	1.32
Relative biases (%)			2.33	0.46	0.22	0.95	1.26	1.51
900	3.5	5.20	12.08	139.66	74.18	79.61	85.93	87.64
Target values			12.12	139.79	74.94	80.54	86.42	88.55
Absolute viases			0.04	0.13	0.76	0.93	0.49	0.91
Relative biases (%)			1.43	0.09	1.04	1.16	0.57	1.03
800	3	5.20	13	137.41	77.69	81.58	87.55	88.59
Target values			13.04	137.54	78.45	82.51	88.04	89.50
Absolute viases			0.04	0.13	0.76	0.93	0.49	0.91
Relative biases (%)			1.43	0.09	1.04	1.16	0.57	1.03

**Table 5:** Process parameter settings for the scale-up strategy of high-shear wet granulation.

Process Parameters	Scale	
	Lab	Pilot
Used granulator	P1	P6
Granulator capacity (L)	1.23	6.05
Batch size (g)	475	2375
Fill level (%)	38.6	39.2
Agitator speed corresponding to agitator tip speed of 6.0 m/s	800 rpm	480 rpm
Massing time (min)	3	3
Spray rate (ml/min)	5.2	5.2

**Table 6:** Validated results for IQAs and CQAs in the scale up.

**Quality Attributes**		**Scale**		**Absolute Biases**	**Relative Biases (%)**
		**Lab**	**Pilot**		
**IQAs**	Granule hardness (N)	2.17	2.25	0.08	3.69
	Carr’s index	13.12	13.45	0.33	2.52
	Granule size (μm)	264.23	275.45	11.22	4.25
	Granule true density (g/cm^3^)	1.453	1.457	0.004	0.28
	Granule bulk density (g/mL)	0.476	0.479	0.003	0.63
	Granule FE	175	173	2	1.16
CQAs	Tensile strength (N/cm^2^)	137.55	138.25	0.70	0.51
	% dissolution at 5 min	78.54	79.15	0.61	0.78
	% dissolution at 10 min	82.55	82.96	0.41	0.50
	% dissolution at 15 min	88.14	88.35	0.21	0.24
	% dissolution at 30 min	89.52	90.08	0.56	0.63
	Friability (%)	0.12	0.11	0.01	9.09
	Content uniformity (%)	0.78	0.74	0.04	5.41
	Assay (%)	101.43	100.78	0.65	0.64
